# Lay knowledge and management of malaria in Baringo county, Kenya

**DOI:** 10.1186/s12936-016-1542-9

**Published:** 2016-09-21

**Authors:** Edna N. Mutua, Salome A. Bukachi, Bernard K. Bett, Benson A. Estambale, Isaac K. Nyamongo

**Affiliations:** 1 Institute of Anthropology, Gender and African Studies, University of Nairobi, P. O. Box 30197, Nairobi, 00100 Kenya; 2 International Livestock Research Institute, P. O. Box 30709, Nairobi, 00100 Kenya; 3 Jaramogi Oginga Odinga University of Science and Technology, P. O. Box 210, Bondo, 40601 Kenya

**Keywords:** Lay management, Malaria, Emetics, Purgatives, Gender, Non-compliance, Artemether-lumefantrine

## Abstract

**Background:**

Malaria, a disease caused by protozoan parasites of the genus *Plasmodium* and transmitted by female anopheline mosquitoes, is a major cause of morbidity, mortality and loss in productivity in humans. Baringo County is prone to seasonal transmissions of malaria mostly in the rainy seasons.

**Methods:**

This cross-sectional study used a mixed methods approach to collect data on knowledge and lay management of malaria. A questionnaire survey was administered to 560 respondents while qualitative data was collected through 20 focus group discussions in four ecological zones covering Baringo North, Baringo South and Marigat sub-Counties of Baringo County. Analyses were done through summary and inferential statistics for quantitative data and content analysis for qualitative data.

**Results:**

The study communities were knowledgeable of malaria signs, symptoms, cause and seasonality but this biomedical knowledge co-existed with other local perceptions. This knowledge, however, did not influence their first (p = 0.77) or second choice treatments (p = 0.49) and compliance to medication (p = 0.84). Up to 88 % of respondents reported having suffered from malaria. At the onset of a suspected malaria case community members reported the following: 28.9 % visited a health facility, 37.2 % used analgesics, 26.6 % herbal treatments, 2.2 % remnant malaria medicines, 2.2 % over the counter malaria medicines, 1 % traditional healers and 1.8 % other treatments. Nearly all respondents (97.8 %) reported visiting a health facility for subsequent treatments. Herbal treatments comprised of infusions and decoctions derived from roots, barks and leaves of plants believed to have medicinal value. Compliance to conventional malaria treatment regime was, however, identified as a challenge in malaria management. Quick relief from symptoms, undesirable qualities like drug bitterness and bad smell, undesirable side-effects, such as nausea and long regimen of treatment were some of the contributors to non-compliance. Men and women exhibited different health-seeking behaviours based on the cultural expectations of masculinity, femininity, gender roles and acceptability of health services.

**Conclusions:**

While knowledge of malaria is important in identifying the disease, it does not necessarily lead to good management practice. Treatment-seeking behaviour is also influenced by perceived cause, severity of disease, timing, anticipated cost of seeking treatment and gender, besides the availability of both traditional and conventional medicines.

## Background

Impoverished communities in marginal areas of malaria endemic countries face the greatest risk of malaria infection and limited access to curative care [1]. These communities are mainly located in sub-Saharan Africa (SSA) which accounted for 88 % of malaria cases and 90 % of malaria deaths in 2015 despite a global decline of 37 % between 2000–2015 [2]. The risk of malaria among impoverished communities is projected to increase due to climatic changes caused by global warming [3]. These changes include temperature increase and change in rainfall patterns which influence malaria seasonality, vector and parasite development, length of transmission periods and geographic distribution [4].

In Kenya, malaria, a leading cause of morbidity and mortality, accounts for 30–50 % of in-patient and 20 % of out-patient morbidity cases, resulting in a projected loss in productivity of 170 million working days per year besides 20 % of deaths among children <5 years [5]. In Baringo county, where this study was conducted, malaria accounts for 11.8 % of the outpatient cases recorded [6]. The county falls under the seasonal malaria transmission zone which is associated with periodic amplification of morbidity in the wet season prompted by limited immunity in inhabitants [7]. The increased number of malaria cases occurs against the backdrop of sub-optimal performance in health facilities due to structural problems/weaknesses such as under-staffing and inadequate medical equipment and sparsely distributed health facilities (the average distance patients travel to health facilities is 15 kilometres) [8]. These challenges leave communities to identify and manage the disease largely on their own.

Malaria research in Baringo county is skewed towards malaria vectors and vector control [9–13] and not disease management at household level. An earlier study in Marigat Division of the same county, showed that people were knowledgeable of malaria signs and symptoms, and used both traditional and conventional methods in managing the disease [14]. Traditional methods involved treatment with medicinal plants while conventional methods utilized analgesics and anti-malarials [14]. This study links lay knowledge and management of malaria in Baringo county to individual health outcomes.

## Methods

### Study area

**Fig. 1:**
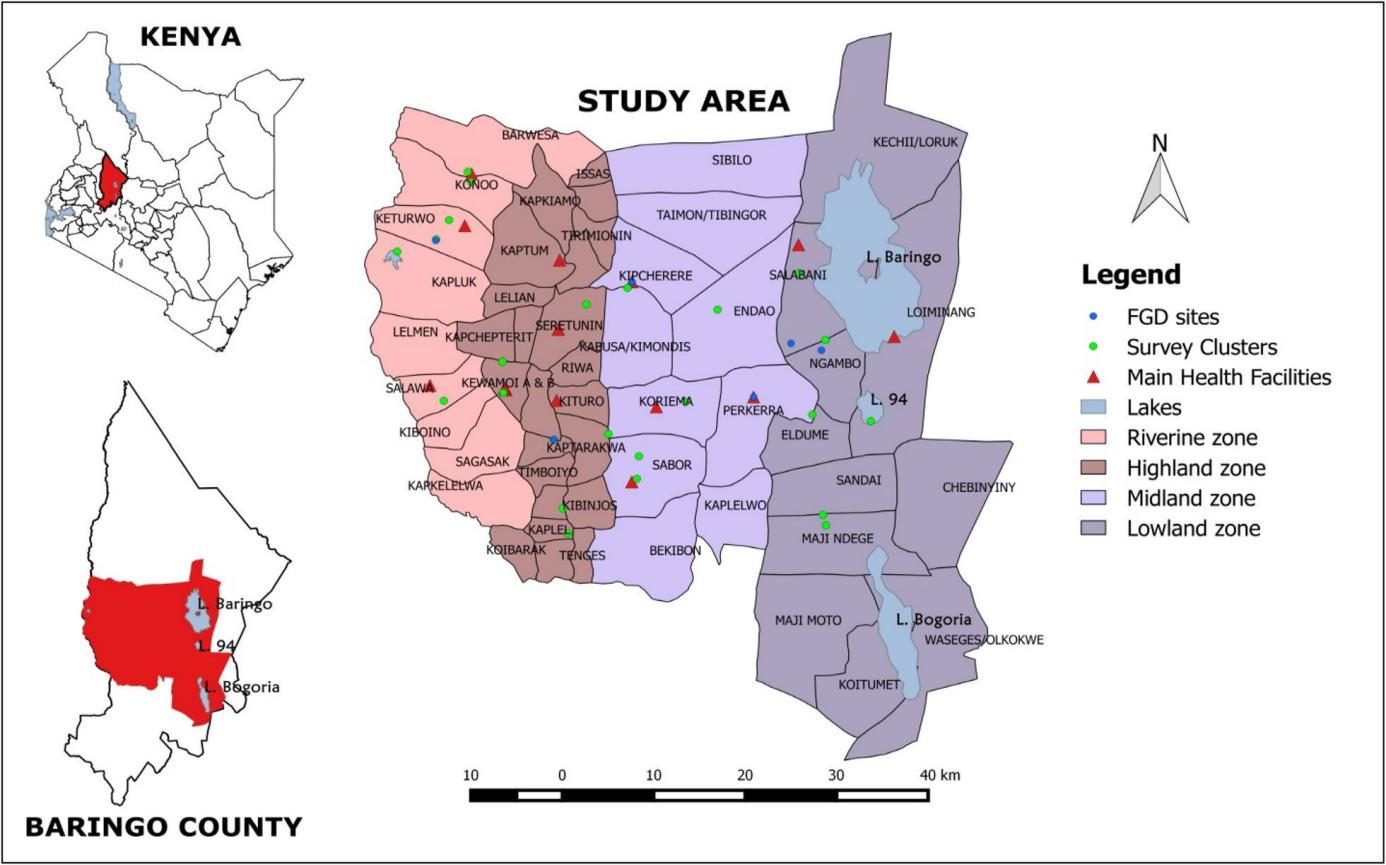
Map of Baringo County showing the study site

The study covers four ecologically diverse zones extending over three sub-counties in Baringo County namely; Baringo Central, Baringo North and Marigat. Dominant communities in the region are the Tugen and Ilchamus. The sites where the different data were collected and the distribution of main health facilities are shown in Fig. 1.

### Study design, sampling and data collection

A cross-sectional community-based study used a sequential mixed methods approach to generate data for this study. Qualitative data was collected prior to the quantitative data and its findings informed the questionnaire design. Quantitative data was collected through a household survey that targeted 560 individuals (260 males and 300 females) drawn from the four ecological zones. Each zone was divided into clusters and systematic sampling was used to select respondents. Qualitative data was collected through 20 focus group discussions (FGDs) (10-male only groups and 10-female only groups) using a semi-structured guide. A total of 164 persons, 76 men and 88 women, who were chosen based on whether they had lived in the area at least through one malaria transmission season and personal experience with malaria, participated in the FGDs. Prior to conducting data collection, the survey questionnaire was pretested with 40 respondents in three areas which shared similar characteristics with the sampled sites and were excluded from the main survey. Necessary amendments were made to the survey tool before data collection. Data was collected by Tugen and Ilchamus speaking enumerators. The FGD guide was pretested in two FGDs comprising exclusively of men or women to test for appropriateness of questions, participatory activities and duration. Relevant adjustments were made prior to actual collection. The discussions lasted between 1–1.5 h.

### Data management and analysis

The survey data was entered and cleaned in CSPro version 6.1 and exported to SPSS version 22 and Stata version 13.1 for analysis. A binary logistic regression model was fitted to test the association between respondents’ overall knowledge of malaria and demographic characteristics. Variance inflation factors were computed to check multicollinearity. Le Cessie-van Houwelingen-Copas omnibus test was used to assess goodness of fit of the model. The overall knowledge of malaria among respondents was determined through 24 questions on malaria cause, signs and symptoms, severity and effects on children and expectant women. Pearson’s Chi square tests of independence were conducted to determine the relationship between respondent knowledge of malaria levels with levels of education and the sub-counties from which they came. Fischer’s exact tests were also conducted to test the relationship between sex and the treatment actions respondents reportedly took when they were sick with malaria and their ability to wait for medical treatment if services delayed. Additional Fischer’s tests were conducted with respondent levels of knowledge of malaria. A significance level of 5 % was used for hypotheses testing.

All FGDs were recorded and notes taken for back-up purposes. The audio files were transcribed verbatim into English by scribes fluent in Swahili and Tugen or Ilchamus languages. Scripts were verified through listening to audio files and comparing with the transcripts. The notes were used for further validation. The cleaned data were coded into emergent themes with NVivo version 10 and analyzed using the content analysis method. During each FGD, pairwise ranking exercises were conducted to determine relative severity of malaria signs/symptoms as perceived by community members.

### Ethical considerations

This research received both national and the World Health Organization (WHO) ethical clearance reference P70/02/2013 and Protocol ID B20278, respectively. Prior to conducting the study, written consent was sought and obtained from all respondents and discussants all of whom were of consenting age (between 18 and 89 years).

## Results

### Participants’ demographic characteristics

A total of 560 respondents (47.5 % male and 52.5 % female) participated in the questionnaire survey. Most respondents were aged between 27–35 years (26.3 %), Christian (99 %), and had primary education (52 %). Nearly three in four (78.4 %) were in monogamous unions, 10.5 % in polygamous ones while the remaining (11.1 %) were single. Their main livelihood activities were crop farming (47.5 %) and livestock farming (20.2 %).

### Knowledge of malaria signs, symptoms and their severity

**Fig. 2:**
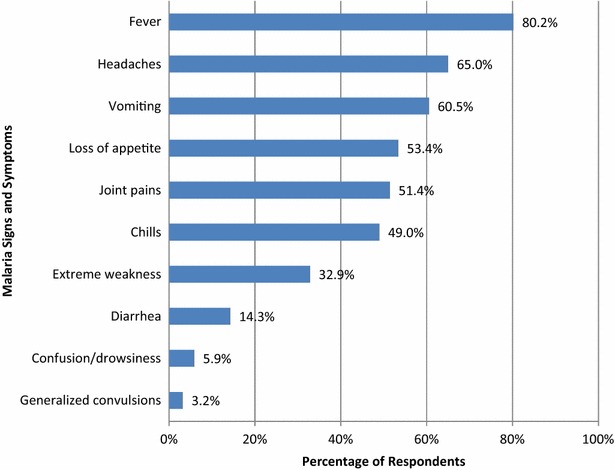
Malaria signs and symptoms (multiple responses)

The study assessed the level of knowledge participants had on malaria, which is locally known as “*esee*” and “*ntikana*” among the Tugen and Ilchamus, respectively. Respondents identified various combinations of malaria signs and symptoms, which comprised of fever (80.2 %), headache (65 %), vomiting (60.5 %), loss of appetite (53.4 %), joint pains (51.4 %), chills (48.8 %), weakness (37.2 %), diarrhoea (14.3 %) and drowsiness (5.9 %) as summarized in Fig. 2. These findings were further reinforced in FGD data. In all discussions, fever, headache, vomiting, loss of appetite, joint pains, weakness, diarrhoea and dizziness/drowsiness were identified as the main signs and symptoms of malaria.

**Fig. 3:**
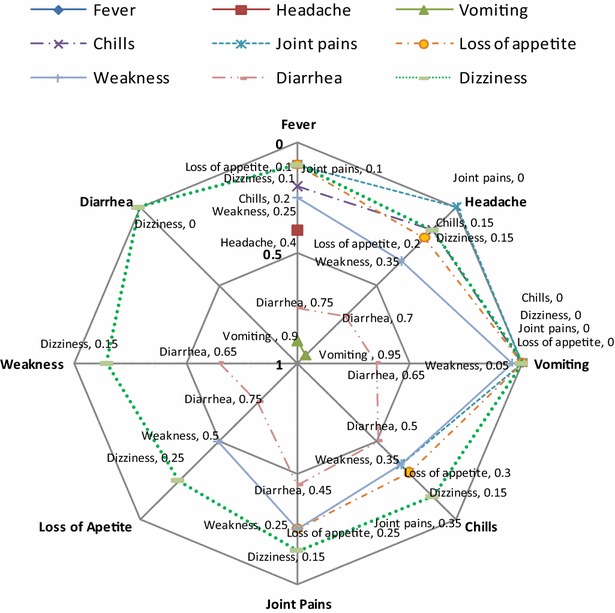
Aggregate pairwise ranking of malaria signs and symptoms

Pairwise ranking exercises were conducted in each of the FGDs to determine the relative severity of each sign or symptom (Fig. 3). They produced the following ranking in decreasing order of severity: vomiting, diarrhoea, fever, headache, body weakness, loss of appetite, joint pains, chills and dizziness/drowsiness. In Fig. 3, the signs and symptoms judged to be most severe are located towards the centre of the radar chart and have a relative weight score closer to 1 while those that are less severe are located towards the periphery and have scores closer to 0. For example, vomiting had the highest relative weight at 0.95 while dizziness had the lowest (0) relative to diarrhoea and vomiting compared to other signs and symptoms. There was no gender difference in the malaria signs and symptoms identified and perceived severity.

### Causes of malaria

**Fig. 4:**
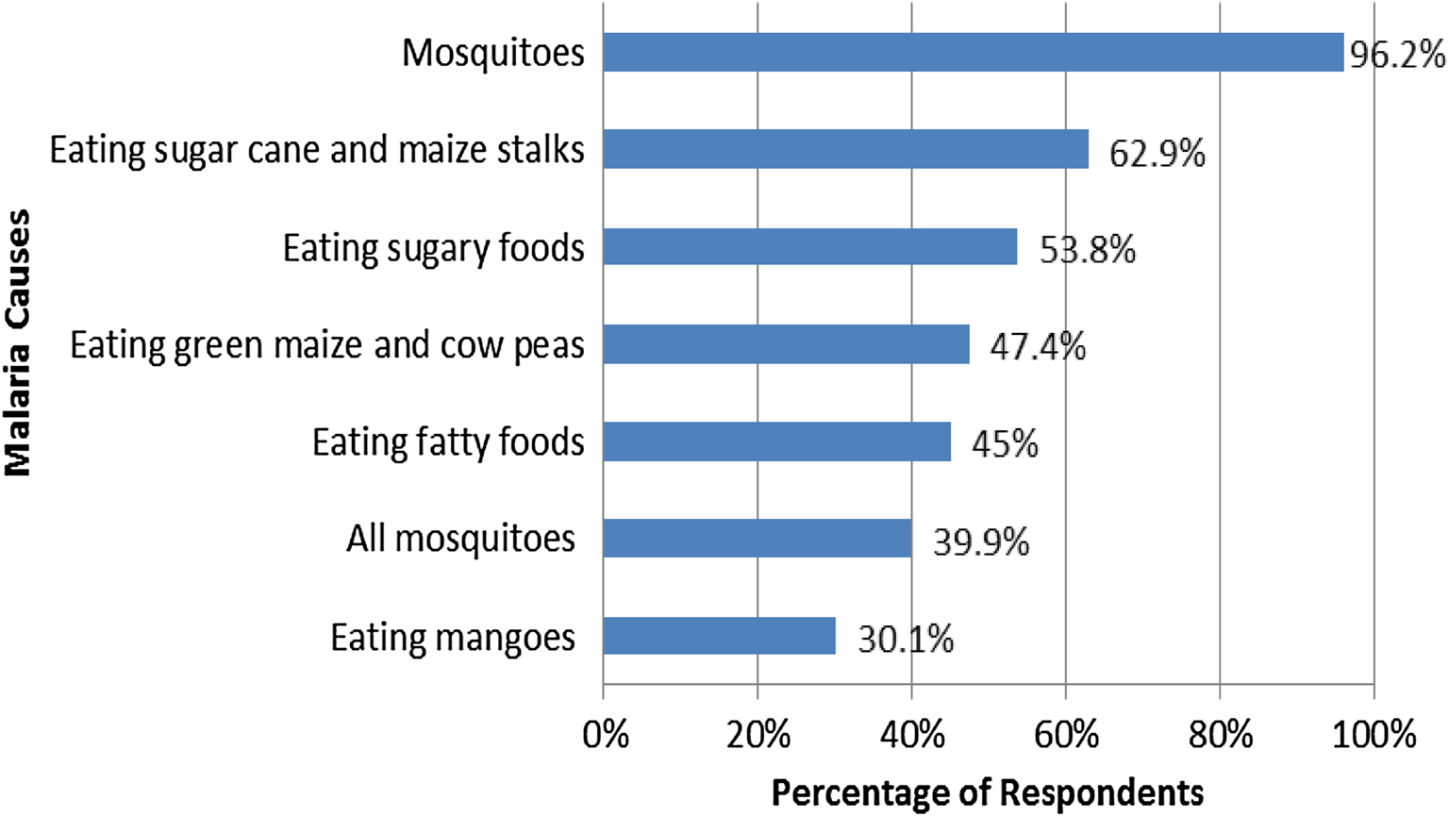
Causes of malaria (multiple responses)

The mosquito was identified by 96 % of the surveyed respondents as a cause of malaria (Fig. 4). However, 40 % believed that all mosquitoes could “cause” malaria. The knowledge that not all mosquitoes transmit malaria was statistically significant and had a positive association with education among demographic characteristics (χ^2^ = 44.01, df = 3, p = 0.000). In addition, there was consensus in all FGDs that mosquitoes transmitted malaria. Discussants in eight FGDs (out of the 20) identified the female *Anopheles* as the disease “causing” mosquito, three groups stated that the malaria “causing” mosquito is female but could not isolate the species, two groups identified the species but could not tell whether it is male or female and seven groups identified the mosquito but could neither specify the species nor the sex. Only in eight FGDs did discussants state that not all mosquitoes had the capacity to transmit malaria.

These views are captured in the following excerpts:

*‘You know our problem is that we assume all types of mosquitoes are the same. But I heard a certain type called* Anopheles *a long time ago in school…. When I’m bitten at night by a mosquito, how do I know that this is* Anopheles *or not?’* Male discussant, Kipcherere 4.

‘*The female* Anopheles *mosquito causes malaria. It is drawn this way*, [demonstrates a 45° angle]. *Its legs look like this* [as she draws] *45°. It stays like this, even when it wants to bite, its legs just draw back that way. If it wants to bite, its legs are raised to a 45° angle. They appear to be standing at an angle of 45°.’* Female discussant, Borowonin 1.

‘[Malaria is caused by] *that female one which doesn’t make noise. It just bites and sucks blood and the following day you feel a bitter taste in your mouth*.’ Female discussant, Salabani 3.

In addition to mosquitoes, it was reported that malaria is caused by consumption of mangoes, fatty foods, sugary foods, green maize and cow peas, sugar cane and maize stalks were widespread. These causes were negatively associated with education: consumption of fatty foods (χ^2^ = 14.87, df = 3, p = 0.002); sugary foods (χ^2^ = 17.17, df = 3, p = 0.001); sugar cane and maize stalks (χ^2^ = 13.26, df = 3, p = 0.004); and green maize and cow peas (χ^2^ = 13.78, df = 3, p = 0.003). Consumption of fatty and sugary foods were the main food related causes identified in 8 and 7 FGDs, respectively. Local communities held the perception that consumption of such foods caused an increase in the amount of bile “*ngw’one*”(Tugen)/“*lodua*”(Ilchamus) in the body thus triggering the occurrence of malaria as exemplified in the following excerpts.

‘*Malaria has become common nowadays because people take a lot of foods rich in sugar which increase bile in the body, after a few days it becomes too much and causes malaria.’* Male discussant, Borowonin 4.

*‘If you eat a lot of fats, it becomes bile and then it becomes malaria.’* Male discussant, Kipcherere 3.

In addition to foods and mosquitoes, water was identified as another key cause of malaria in 15 FGDs. Consuming water from the lowlands of Baringo (6), water contaminated with mosquito eggs (5) or other dirt (5) and stagnant water (4) were reported as causes of malaria as shown in the excerpts below.

*‘There used to be a belief, especially for us who live here* [in the highlands]*, that if we go to lowland areas like Marigat and Lodwar, or if someone goes there and comes back they will have malaria directly. I don’t know why. I used to think that in that place the sun is very hot and when you are hit by that sun and you drink their water, when you come back here, you get malaria directly’*. Female discussant, Borowonin 1.

*‘Also, people in the past never knew malaria was brought by mosquitoes. They thought it was brought by water. So when somebody was going somewhere, they took some soil and when they reached there* [where they were going]*, they asked for water and mixed a little with the soil and consumed. They did that thinking it prevents malaria because water from other places can affect you. They used to believe that but still they got malaria’.* Male discussant, Kipcherere 4.

### Malaria seasonality

There was consensus in the FGDs that malaria cases could occur all year round but more cases were reported to occur during the rainy season. Living close to permanent water bodies was also identified as a contributor to malaria infection in the dry season.

‘*During the dry season like now* [March], *the prevalence is low but during the rainy season we experience a lot of malarial cases’.* Female discussant, Perkerra 2.

*‘You know, those people who live near rivers can get malaria. For example, if you see in the hospital, those who have malaria during this dry season* [July] *are those people who come from down there, next to the Kerio river’.* Male discussant, Litein 3.

### Overall knowledge of malaria

The overall knowledge of malaria among respondents was determined through 24 questions on malaria cause, signs and symptoms, severity and effects on children and expectant women. The mean score was 17 (s.e. = ± 0.144). A score of <17 was classified as having moderate knowledge while ≥17 was classified as highly knowledgeable. Up to 45.5 % (95 % CI 41.4–49.8) of respondents had moderate knowledge while 54.5 % (95 % CI 50.2–58.6) were highly knowledgeable. A binary logistic regression model, whose goodness of fit was confirmed using the le Cessie-van Houwelingen-Copas omnibus test (χ^2^ = 57.12, df = 18, p = 0.000), was fitted to test the association between overall knowledge of malaria and respondents’ demographic characteristics. These included sex, marital status, household type, level of education and the main livelihood activity practiced. Of the predictor variables, sex, marital status, household type, education and livelihood activity were statistically significant (Table 1). The odds of women being highly knowledgeable were 1.51 times more likely than that of men. Respondents from monogamous marriages were 0.28 times less likely to be knowledgeable compared to those who had never been married. Respondents from de jure female-headed households, were 0.24 times less likely to be knowledgeable than those from male-headed households. Respondents with secondary and tertiary education were 1.99 and 3.21 times more likely to be knowledgeable than those with no education. The level of education and knowledge of malaria had a positive and significant relationship (χ^2^ = 25.54, df = 3, p = 0.001). This shows that the higher the level of education, the more the knowledge on malaria a respondent had. Across the sub-counties, there was no difference in the levels of knowledge (χ^2^ = 5.10, df = 2, p = 0.08). Respondents whose main livelihood activity was livestock farming were less likely to be knowledgeable than those who practiced crop farming by 0.46 times.

**Table 1:** Logistic regression model analyzing the association between knowledge and demographic characteristics

Logistic regression				Number of obs = 560	
				LR χ^2^ (18) = 57.12	
Log likelihood = −357.36707				Prob > χ^2^ = 0.000	
				Pseudo R^2^ = 0.07040	
Knowledge	Odds ratio	Std. error	z	p > |z|	95 % CI
*Sex*					
Female	1.506	0.312	1.97	*0.048*	1.003–2.261
*Marital status*					
Married monogamous	0.278	0.184	−1.94	*0.053*	0.076–1.015
Married polygamous	0.322	0.228	−1.60	0.110	0.081–1.290
Separated	0.960	0.890	−0.04	0.965	0.156–5.905
Widowed	0.624	0.446	−0.66	0.510	0.154–2.533
*House type*					
Male only, no female adult	0.797	0.394	−0.46	0.647	0.303–2.101
Female only, no male adult	0.245	0.116	−2.97	*0.003*	0.097–0.619
*Age*	0.990	0.007	−1.36	0.173	0.977–1.004
*Education*					
Primary	0.727	0.199	−1.16	0.245	0.425–1.244
Secondary	1.990	0.681	2.01	*0.044*	1.017–3.891
Tertiary	3.209	1.633	2.29	*0.022*	1.183–8.702
*Main livelihood activities*					
Livestock farming	0.462	0.111	−3.21	*0.001*	0.288–0.741
Self-employed service delivery	0.627	0.233	−1.26	0.208	0.303–1.297
Self-employed goods delivery	0.657	0.209	−1.32	0.186	0.353–1.225
Wage employment	0.733	0.250	−0.91	0.363	0.376–1.431
Salaried employment	1.069	0.503	0.14	0.888	0.425–2.690
None	1.310	1.414	0.25	0.803	0.158–10.863
Other	0.423	0.335	−1.08	0.278	0.090–2.001
_cons	6.844	5.539	2.38	0.017	1.401–33.433

Reference groups: sex-male, marital status-single (never married), household type-male headed with spouse, education-none, main livelihood activity-crop farming

### Treatment options and pathways

Up to 87.9 % of respondents reported having suffered from malaria in their lifetime. At the onset of a suspected malaria case, 37.2 % of respondents (n = 492) reportedly took locally available variants of paracetamol or aspirin, 28.9 % visited a health facility for treatment, 26.6 % used herbal treatments, 2.2 % took remnant malaria medication while 2.2 % took malaria medicines bought from a chemist. If the disease became more severe, 97.8 % of respondents (n = 492) sought treatment in a health facility. Of these, 81.9 % were tested before treatment with artemether-lumefantrine (AL) the last time they had malaria. Efficiency in treatment was the most desirable AL quality as identified by 84.5 % of respondents. However, to get cured, they had to bear with some undesirable characteristics/side-effects of the same medicines. These included bitterness (31.3 %), bad smell (30.5 %), nausea (27.2 %) and long regime of treatment (5.1 %). Only 10.2 % of respondents reported that these undesirable qualities had ever hindered them from complying with malaria treatment regimen. Further, 93.7 % of respondents (n = 492) reported that they completed their last AL dose. Fischer’s exact tests were conducted on the level of knowledge of malaria and treatment actions taken by sick individuals but the relationship was not statistically significant (Table 2).

**Table 2:** Fischer’s exact tests on the relationship between level of knowledge, sex and treatment actions

Treatment actions		N	Level of knowledge	Sex
			P value	P value
1	1st choice treatment	492	0.772	0.307
2	2nd choice treatment	492	0.494	1.000
3	Testing for malaria	488	0.366	0.549
4	Completing AL doses	489	0.840	0.688
5	Not completing AL doses due to their undesirable qualities	490	0.373	0.024
6	Not seeking services in the nearest facility because of slow delivery	560	0.822	0.001

There was consensus among discussants that people followed different approaches in managing malaria. These included taking analgesics, herbal medicines, remnant malaria medicines or visiting a health facility. As reported in all FGDs, paracetamol was mainly taken to alleviate pain associated with the disease before further treatment was sought. Herbal medicines were used either to decelerate progression of malaria prior to seeking medical treatment or treat it. Various medicinal plant leaves, roots or barks with emetic or purgative qualities were prepared into infusions or decoctions then consumed 1–3 times a day for 1–3 days. The perception that healing from malaria occurred when the body is cleansed of excess bile caused by consumption of fatty or sugary foods through the use of purgatives and emetics, a phenomenon locally referred to as “*kecheru ngw’one*”(Tugen)/“*aitei lodua*”(Ilchamus) was widespread. The use of herbal medicine seemed to be more popular with older community members than younger ones as shown in the following excerpts:

*‘To add to what the man has said, nowadays people have gone to school. You will find that the digital* [young people] *don’t like herbal medicines so much but the analogue* [old people] *continue using them’*. Male discussant, Salabani 1.

*‘You see these medicinal plants, not everyone knows them. Not everyone knows that they are medicinal. You know we stay in town, near the hospital. They* [people] *are close to the hospital and maybe they do not know those herbs so they just have to go to the hospital’*. Male discussant, Perkerra 1.

The treatment decisions were influenced by the rate of disease progression, availability of treatment option, cost of seeking treatment, timing illness onset and distance to preferred health facility. If an individual recovered from the perceived malaria after the first choice treatment, no further treatment was sought. If the disease persisted, the second choice of treatment was sought in a health facility where treatment with AL was initiated. However, focus group discussants were of the opinion that non-compliance was widespread with the leading cause being quick relief from signs and symptoms, a quality linked to the efficiency of AL, in 18 of the FGDs. Undesirable malaria drug qualities like bitterness (13), bad smell (8) or the drug’s side-effects such as causing vomiting (12) or nausea (10) and many tablets to swallow (3) also contributed to non-compliance as shown in the excerpts below.

*‘In this area, if you take the tablets in the morning, because of the hot sun you will do nothing the whole day. You will feel bad when you go to the sun. You smell of that medicine. Your whole body and sweat is like that medicine so some people prefer taking the medicine in the night and skip taking them in the morning. I take the malaria medicines in the evening only. I take* [the medicine] *for six days though it is not correct to skip other times’.* Female discussant, Salabani 4.

*‘When I was sick I took eight tablets from the first day’s dose, then my daughter who’s in university got ill and we could not get more medicine. I gave her the rest, we shared’.* Male discussant, Litein 1.

Remnant malaria medication was kept and used as first choice treatment when another case of perceived malaria occurred with or without confirmation of expiry.

*‘They [people] check the expiry date* [of the remnant malaria medication], *if it is past, it’s not supposed to be taken. They then visit the hospital. But if it is not yet expired they use. If they feel well they will not go to the hospital but if they don’t they will’.* Male discussant, Borowonin 3.

*‘But some people don’t check for the expiry date of remnant malaria medicines. You have said that because you know that one should check but what of those that do not check the expiry date?’* Male discussant, Perkerra 1.

### Gender differences in health-seeking behaviour

Gender issues manifested in health-seeking behaviour among men and women with suspected malaria. There were no gender differences in the first (Fischer’s exact test p = 0.307) and second (p = 1.000) choice treatments, testing for malaria (p = 0.549) and completing AL doses (p = 0.688) (Table 2). However, there was a statistically significant relationship between sex and having failed to seek for medical services due to long waiting periods in health facilities (χ^2^ = 11.442, df = 1, p = 0.000) and not completing malaria medicines due to their undesirable qualities (Fischer’s exact test p = 0.02). More men than women failed to seek medical treatment if services were slow while more women than men had ever failed to comply with malaria treatment regimen because of the undesirable qualities/side-effects (Table 2). In the FGDs, discussants similarly identified key gender differences in health-seeking patterns. In 14 FGDs, there was consensus that women sought medical treatment sooner than men. For women, delays in seeking treatment translated to delays in recovery and declined ability to meet their reproductive and productive roles. In these communities, men’s and women’s household chores were clearly defined and men rarely substituted women’s labour.

*‘You know women have a lot of work so if you get very sick, you start saying, this homestead, who will manage it so you just say I should go to see the doctor’.* Female discussant, Borowonin 1.

*‘The family depends upon the woman. The children and husbands also depend on the women. Also all the house chores depend on her. So she will have to go to the hospital so as to get well fast’.* Female discussant, Perkerra 2.

It was stated that men (16 FGDs) delayed in seeking treatment and were likely to present with severe cases. The delays were mainly associated with the belief that they had to show stoicism failure to which they would be assumed to be weak (12 FGDs).

*‘It is a norm that a man must persevere. So he* [sick man] *does not want to show weakness even that time when he is almost dying…. He has been taught that in our culture you should not live in a bad manner* [showing weakness]*. You have to stay strong. If you are a man, be a man and live like a man and not like a woman’.* Female discussant, Kipcherere 2.

Fear of testing for malaria, which was assumed to be done simultaneously with HIV testing (10) and dislike for waiting for services in hospital queues (3) added to the deterrents. The excerpts below exemplify this behaviour:

*‘They [men] think if they go to be tested for malaria, they will be tested for HIV also’*. Female discussant, Perkerra 2.

*‘If you are in doubt about your HIV status, people* [men] *fear to have their blood drawn for testing, but if you know you are okay there is no problem’.* Male respondent, Borowonin 4.

*‘There are those men who are patient, you can’t compare me with another man. Some men don’t want to be in the hospital queues. …. So someone may say* [that] *they are wasting time* [in queues]’. Male discussant, Borowonin 3.

*‘When it comes to men, like the old men, they don’t go to the hospital because they wonder ‘who is going to queue?’ There are those who don’t go completely and God still loves them, they still get better’.* Female discussant, Borowonin 2.

In addition, men had greater control of financial resources and thus were in a better position to make firm decisions regarding their own treatment without the need to consult. This unequal ownership of resources is captured by one male and two female FGD discussants below:

*‘If they* [the sick person] *are supposed to go to the hospital, it is the man/father/husband who contributes the money to take them’.* Male discussant Salabani 2.

*‘When you have a sick person at home suffering from malaria or any other disease, it is the work of the woman to take care of that person. The men help by providing money to take that person to the hospital’.* Female discussant Perkerra 3.

*‘There is a big difference* [in asset ownership] *because a man is always the head of the family and the woman should always be under him’.* Female discussant Soruro 2.

Therefore, due to fear of getting tested for HIV in addition to malaria tests, long time it took to get services because of the long queues in government health facilities and their greater control of financial resources, men were more likely to buy medicines from chemists or visit private health facilities whose services were quicker.

## Discussion

More than half of the respondents identified at least five malaria signs and symptoms, mainly fever, headache, vomiting, loss of appetite and joint pains. The most recognizable symptom was fever (80.2 %) while the least was convulsions (0.8 %). These findings concur with other studies on community knowledge of malaria signs and symptoms (see, in Tanzania [15], Uganda [16], Ethiopia [17] and Ghana [18]) where fever is cited as the most widely known symptom of malaria. Convulsions as a sign of severe malaria has been identified as less known in previous studies (in Tanzania [15]; Uganda [16]). Contrary to findings from the present study, in South Africa a study reported headache as the most cited malaria sign while fever was poorly known [19]. Another study in Ethiopia found that chills were the most cited malaria sign followed by fever [20]. Although the respondents identified the main signs and symptoms of malaria, the small proportion of individuals who recognize convulsions suggests a critical knowledge gap that should be addressed.

While the identified signs and symptoms may signify malaria, it is important to recognize that malaria shares symptomatology with other diseases such as typhoid [21] and brucellosis [22] in which patients experience headaches, fever, vomiting, diarrhea, and body weakness among other signs and symptoms. To the lay person, differentiating between the diseases is difficult. Thus, there is a possibility that the diseases assumed to be malaria at community level may turn out to be other infections upon biomedical testing. In cases where malaria is diagnosed clinically, there lies further risk of over diagnosis and misuse of anti-malarials fueled by the tendency to associate these signs and symptoms with malaria rather than a group of disease [23].

Local perceptions on causes of malaria coexist together with biomedical knowledge, a situation not unique to Baringo. Besides mosquitoes, other causes have been identified across Africa [15, 17, 18, 24]. This reflects a knowledge gap between biomedical and local perceptions of malaria etiology and carries potential for influencing malaria treatment choices. Indeed, this was the case in Baringo where the use of plant-based emetics and purgatives reported in this study was driven by the perception that malaria was caused by consumption of fatty or sugary foods which triggered an increase of bile levels in the body resulting in malaria. Similar findings have been documented among the Marakwet [25], Nandi [26], subtribes of the Kalenjin and the Maasai [27] and Samburu [28] subtribes of the Maa communities. The Tugen and Ilchamus are sub-tribes of the Kalenjin and the Maa communities, respectively. The fact that these local perceptions are widespread among members of loosely related communities (of whom the Tugen and Ilchamus are members) suggests that these perceptions are not random and irrational but could be grounded in local cosmologies.

The treatment patterns displayed by ailing persons are influenced by a multiplicity of factors. An earlier study on health-seeking behaviour of malaria patients in Marigat area in Baringo county found they first sought medical care in public health facilities whose services were offered free of charge and if treatment failed, they went to private health facilities or took herbal medicines [14]. Another study conducted among the Abagusii of Kenya found that persons sick with malaria cut on costs by first treating themselves at home and only went to health facilities if the disease persisted [29]. The first and second choice treatment patterns followed by respondents in this study were influenced by perceived severity of illness, options available for treatment, anticipated cost of seeking medical care, proximity to health facility of choice and illness occurrence timing. In the current study, two-thirds of the respondents attempted treatment at home and only went to a health facility if the case persisted. The relationship between their level of knowledge of malaria and the actions taken in seeking conventional medicine were not statistically significant indicating that having knowledge alone did not necessarily influence health-seeking behaviour.

Additional factors such as local understanding of malaria, perceived severity, perceived benefits of seeking healthcare, proximity to health facilities, availability of medicines and availability of money to cover treatment costs also influence health seeking behaviour [30]. In this study, men preferred to seek healthcare services where they were delivered within a short time; an expectation not likely to be met in public health facilities. Discussants also reported that more men than women were not likely to seek health services in a facility for fear that they would be simultaneously tested for malaria and HIV, a concern also documented in western Kenya in a study on mass testing and treatment of malaria [31]. On the other hand, more women reported not having completed their malaria medication owing to its adverse effects. The culturally acceptable threshold of disease endurance before disruption of normal activities permissible for men and women also influences access to health services [32]. As demonstrated in this study, men were culturally expected to exercise more endurance than women and hence presented in health facilities later. This practice carried the risk of delaying men’s access to health services and consequently presenting with severe cases.

Plants remain important sources of medicine and have been used to treat human ailments since time immemorial. Preferred for their affordability, accessibility and cultural acceptability, herbal medicines offer an additional treatment option in the arid and semi-arid regions of Kenya [33] which have poor infrastructure and health services [34]. However, the use of these medicines is fraught with challenges. Firstly, homemade doses tend to be unstandardized and may effect under or over consumption of active ingredients [33] making them ineffective [35] or causing adverse effects [21] respectively. Secondly, the efficacy of many of the plants used as medicine is scientifically unproven due to insufficient pharmacological research and may interact with prescription medicines [33] Thirdly, the knowledge of the medicinal plants and their uses is passed from one generation to another orally and is not documented hence is at risk of distortion and loss [33]. Lastly, use of traditional medicines simultaneously with conventional malaria medicine may lead to non-compliance to biomedical treatment resulting in sub-optimal parasitological and clinical outcomes [36].

Malaria testing and medicines are provided at no cost in public health facilities in accordance with national guidelines. However, making medicines accessible does not necessarily ensure compliance. After diagnosis and treatment in health facilities, compliance is left to the individual and is largely unsupervised. In this study, the level of compliance to treatment reported in the survey was higher than that reported in the FGDs. This may be due to respondents who know the expected practice reporting compliance to save face and avoid presenting themselves as negligent [37]. However, a systematic review of evidence on ACT compliance showed that cross-sectional household surveys like this one reported higher levels of compliance due to recall bias [38].

Non-compliance to ACT has been linked to a variety of reasons. In Nigeria, it has been linked to bad pill taste, tablet size, treatment duration, administration frequency and cost of medicine [39]. In a study conducted in the Tigray region of Ethiopia, the reasons given for non-compliance were pill bitterness, size (being too big to swallow), quantity, recovery before completing the requisite medication course, forgetfulness, saving medicines for future infections and declining to take the medicines [40]. In Western Kenya, a study on determinants of anti-malarials use associated non-compliance with getting well before completing the treatment course, sharing medicines with someone else and saving the medicine for future use [41]. Another study in western Kenya, focusing on factors associated with non-compliance established that being older, having low education level, low monthly household income and low literacy were contributors [42]. Thus, the reasons given in this study for non-compliance are not unique to Baringo. Instead, they are a cause for public health concern if efficacy of the first line of malaria medicines is to be maintained.

### Study limitations

The study was limited to parts of Baringo central, Baringo north and Marigat and not the six sub-counties of Baringo county. In terms of methodology, the qualitative data presented in this paper cannot be generalized since the participants were chosen purposively and their experiences were very context specific. In addition, in the translation of qualitative data from Turgen, Ilchamus or Swahili language to English, some cultural meanings may have been lost. However, these losses were controlled through the use of scribes knowledgeable in at least one of the local languages and Kiswahili and verification of scripts upon transcription. The findings presented in this paper were based on what research participants reported they did rather than direct observations and verification of what they claimed to do.

## Conclusions

Most community members had a firsthand experience with malaria and could identify signs and symptoms, seasonality and cause. Biomedical knowledge and local perceptions of malaria causality coexist which seem to influence treatments as displayed by community’s use of both conventional and herbal treatments. The co-existence of biomedical knowledge and local perceptions seems to impact on treatment compliance. Therefore, this study recommends raising of public awareness through use of educational programmes tailored to address existing biomedical and local knowledge gaps in order to reduce non-compliance. The education programmes will take into account the low literacy levels by moderating the messages to make them easily comprehensible. However, it must be noted that high levels of awareness at community level will not necessarily result in compliance. Other influencing factors such as cost of seeking service, quality of care and proximity of facilities to the communities need to be addressed also.

## Abbreviations

AL: artemether-lumefantrine
FGD: focus group discussion(s)
SSA: Sub-Saharan Africa
WHO: World Health Organization
